# Editorial: Regulation and mechanism of plant metabolites on hyperuricemia

**DOI:** 10.3389/fnut.2026.1800834

**Published:** 2026-02-17

**Authors:** Yin Wan, Jin Qian, Tao Xiong, Jun Lu

**Affiliations:** 1 State Key Laboratory of Food Science and Resources, Nanchang University, Nanchang, China; 2 Auckland Bioengineering Institute, and School of Chemical Sciences, University of Auckland, Auckland, New Zealand

**Keywords:** gut-kidney axis, hyperuricemia, multi-omics, NLRP3 inflammasome, plant metabolites, uric acid transporters

## Introduction and significance

Hyperuricemia (HUA) has rapidly evolved from a “rich man's disease” to a global public health crisis. According to the latest data from the Global Burden of Disease (GBD) Study 2019 published in 2024, the global prevalence of gout has increased by 63.44% over the past three decades, with high body mass index (BMI) identified as the primary driver (1). Beyond its role in gout, accumulating evidence highlights that elevated soluble uric acid is now recognized as a potent “danger signal” (DAMP) that triggers systemic inflammation and metabolic maladaptation (2).

Despite this growing burden, clinical management remains stagnant. First-line xanthine oxidase (XOD) inhibitors like allopurinol and febuxostat are plagued by severe cutaneous adverse reactions, cardiovascular risks, and renal injury (3). This creates a critical gap: How do we achieve safe, long-term urate management without compromising organ function? This Research Topic, *Regulation and Mechanism of Plant Metabolites on Hyperuricemia*, addresses this gap by shifting the paradigm from “single-target inhibition” to “systemic network regulation.” The seven articles collected here do not merely screen for natural products; they elucidate how plant metabolites remodel the “Gut-Kidney-Immune” axis, offering a mechanistic bridge between traditional botanical wisdom and modern precision medicine.

## Epidemiological insights and structural biology-based discovery

The journey of intervention begins with identifying dietary determinants. In a large-scale analysis of NHANES 2001–2006 data, Chen, Miao et al. revealed a significant inverse association between serum carotenoids (specifically lycopene and α-carotene) and HUA risk. This finding aligns with the “Food is Medicine” concept emphasized by Mozaffarian et al., suggesting that specific phytochemicals can serve as prophylactic agents at the population level (4).

Moving from epidemiology to drug discovery, Chen, Chen et al. employed high-throughput virtual screening to identify XOD inhibitors from *Zanthoxyli Pericarpium*. It is worth noting that Mullowney et al. recently highlighted that integrating computational strategies with experimental validation is revolutionizing the discovery of bioactive natural products (5). Echoing this trend, Chen, Chen et al.'s molecular dynamics simulations provide atomic-level insights into how natural compounds like ZP-30 bind to key metabolic enzymes, placing their work at the forefront of structure-based drug design (SBDD).

Collectively, these studies bridge the gap between macroscopic epidemiological patterns and microscopic molecular mechanisms, exemplifying a comprehensive approach to validating the therapeutic potential of natural products.

## Multidimensional insights into the “Gut-Kidney Axis” and immunometabolism

The most transformative advance in HUA research in the last 3 years is the elucidation of the “Gut-Kidney Axis.” Kasahara et al. demonstrated that gut microbiota-derived metabolites can directly influence renal urate excretion (6). In this Research Topic, Rao et al. advance this frontier by identifying specific *L. reuteri* and *L. brevis* strains that not only degrade nucleosides but also repair the intestinal barrier. Their work confirms that probiotics can serve as “living drugs” to intercept purine absorption, a strategy that complements renal-targeted therapies.

Simultaneously, the role of the NLRP3 inflammasome in urate-induced kidney injury has become a consensus in the field, as systematically reviewed by Luo et al. (7). Contributing to this field, Wang, Zhu et al. demonstrated that Aloe-emodin suppresses the NLRP3/caspase-1 pathway, effectively decoupling high uric acid levels from renal fibrosis. Similarly, Zhao et al. found that *Puerariae lobatae* Radix regulates the “reabsorption-secretion” transporter network (URAT1/OAT1). These findings are particularly significant when viewed alongside the findings by Fan et al. on URAT1 inhibition mechanisms, illustrating that plant extracts can exert effects comparable to synthetic inhibitors but with pleiotropic benefits (8).

Thus, by targeting both the upstream intestinal barrier and downstream renal inflammation, these studies collectively propel the understanding of the gut-kidney axis, offering a holistic immunometabolic framework for future therapies.

## Systems pharmacology and clinical translation

Finally, HUA is a systemic metabolic disorder requiring systemic solutions. Wang, Ding et al. utilized multi-omics and network pharmacology to decode the *Guiling Prescription*, revealing a synergistic mechanism spanning inflammation control and metabolic reprogramming. Bridging the gap to the clinic, Shen et al. presented interim clinical trial results for the *Qifu Huazhuo Formula*. In an era where “Precision Nutrition” is gaining traction, their integration of clinical phenotypes with serum proteomics provides a robust evidence base for personalized Traditional Chinese Medicine (TCM) interventions.

## Conclusion

Collectively, these seven articles demonstrate that plant metabolites are not merely dietary adjuncts, but sophisticated modulators of the Gut-Kidney-Immune network. By targeting the latest molecular checkpoints—from the NLRP3 inflammasome to the gut microbiome—this Research Topic paves the way for a new generation of HUA therapies that are effective, holistic, and safe.
